# Structural Diversities of Lectins Binding to the Glycosphingolipid Gb3

**DOI:** 10.3389/fmolb.2021.704685

**Published:** 2021-07-26

**Authors:** Lina Siukstaite, Anne Imberty, Winfried Römer

**Affiliations:** ^1^ Faculty of Biology, University of Freiburg, Freiburg, Germany; ^2^ Signalling Research Centres BIOSS and CIBSS, University of Freiburg, Freiburg, Germany; ^3^ CNRS, CERMAV, Université Grenoble Alpes, Grenoble, France; ^4^ Freiburg Institute for Advanced Studies (FRIAS), University of Freiburg, Freiburg, Germany

**Keywords:** lectin, glycosphingolipid, globotriaosylceramide, Shiga toxin, valence, bacterial adhesins, carbohydrate

## Abstract

Glycolipids are present on the surfaces of all living cells and thereby represent targets for many protein receptors, such as lectins. Understanding the interactions between lectins and glycolipids is essential for investigating the functions of lectins and the dynamics of glycolipids in living membranes. This review focuses on lectins binding to the glycosphingolipid globotriaosylceramide (Gb3), an attractive host cell receptor, particularly for pathogens and pathogenic products. Shiga toxin (Stx), from *Shigella dysenteriae* or *Escherichia coli*, which is one of the most virulent bacterial toxins, binds and clusters Gb3, leading to local negative membrane curvature and the formation of tubular plasma membrane invaginations as the initial step for clathrin-independent endocytosis. After internalization, it is embracing the retrograde transport pathway. In comparison, the homotetrameric lectin LecA from *Pseudomonas aeruginosa* can also bind to Gb3, triggering the so-called lipid zipper mechanism, which results in membrane engulfment of the bacterium as an important step for its cellular uptake. Notably, both lectins bind to Gb3 but induce distinct plasma membrane domains and exploit mainly different transport pathways. Not only, several other Gb3-binding lectins have been described from bacterial origins, such as the adhesins SadP (from *Streptococcus suis*) and PapG (from *E. coli*), but also from animal, fungal, or plant origins. The variety of amino acid sequences and folds demonstrates the structural versatilities of Gb3-binding lectins and asks the question of the evolution of specificity and carbohydrate recognition in different kingdoms of life.

## Introduction

Glycans are on the surface of all living cells and play a remarkable role in the immune system, cellular signalling, and host-microbe interactions. With nucleic acids, proteins, and lipids, carbohydrates are building blocks, which are by far more complex and faster evolving. The stunning diversity of glycans in branching, length, and linkages is achieved from various monosaccharides assembled by specific enzymes–glycosyltransferases in the ER and Golgi apparatus (Varki et al., 2017). Glycoproteins, proteoglycans, and glycolipids at the cell surface present complex glycoconjugates, and their composition, conformation, and dynamics constitute the glyco-code (Gabius et al., 2004).

Specific proteins, so-called lectins, can decipher the glyco-code. Lectins are sugar-binding proteins recognizing specific structures of carbohydrates. Differently from glyco-enzymes, transporters, or antibodies, lectins tend to have shallow but well-established ligand-binding pockets or grooves to recognize one or few moieties of oligosaccharides at the terminal and subterminal positions, and do not present any catalytic activity (Lis and Sharon, 1998). Lectins are present in all living organisms and can adopt various folds, as illustrated in the Unilectin3D database (Bonnardel et al., 2019). They are generally multivalent, with several carbohydrate recognition domains assembled through oligomerization or tandem repeats along the peptide sequence.

Lectins play a role in various biological functions in self/non-self-recognition, being of first importance in developing interactions of host cells with pathogens. Many microbes, such as viruses, bacteria, parasites, and fungi, use lectins to bind to glycans present on target tissues during the infection process. In many cases, lectins are involved in the adhesion process leading to infection (Sharon, 1996). This provided the basis for developing therapeutic strategies where the lectin itself is targeted as an anti-infectious approach (Chabre et al., 2011; Novoa et al., 2014; Meiers et al., 2019). Plant and fungal lectins are involved in defence mechanisms and the establishment of symbiosis. Moreover, they are very useful for applications in research and technology. Due to their multivalence, many lectins can bind with strong avidity to the glycans presented in multiple copies at the surface of cells (Dam and Brewer, 2010).

Glycolipids represent targets for many lectins, and the binding of lectins may affect their dynamics in membranes. The interactions between lectins and glycolipids have been characterized for a better understanding of biological processes and investigating the dynamics of glycolipids in living membranes. Among them, Gb3, the glycosphingolipid globotriaosylceramide, is utilized by several pathogens as host cell receptor (Lund et al., 1987; Kirkeby et al., 2006; Gallegos et al., 2012; Johansson et al., 2020), and it is also overexpressed in several human cancers. Gb3 plays therefore an essential role in both human health and disease and can be utilized as target for drug delivery approaches (Müller et al., 2017).

This review examines the three-dimensional structures of Gb3-binding lectins to illustrate the structural basis for their carbohydrate-binding affinity and specificity. We will cover Gb3-binding lectins from bacteria, but also from fungi, plants, and animals since they are of potential interest for targeting Gb3. Typical features for Gb3-binding microbial lectins will be investigated. We will describe substrate specificities, folds, binding site topographies, valency, affinity, and intracellular trafficking.

## Background and Importance of the Glycosphingolipid Gb3

Glycosphingolipids (GSLs) are mainly present in the outer leaflet of the plasma membrane (Hakomori et al., 1998; Kasahara and Sanai, 1999; Schnaar and Kinoshita, 2015). They are known to be involved in embryonic development, apoptosis, cell adhesion, intercellular coordination, cell differentiation, signal transduction, and cancerogenesis of multicellular organisms. However, a more precise characterization of the physiological role of GSLs was difficult in the past due to the lack of appropriate tools, such as labelling and detection methods. Among several classes of GSLs, globosides are defined as neutral glycolipids with at least two monosaccharides linked to the ceramide backbone. The carbohydrate moiety of globosides usually includes combinations of D-glucose (Glc), D-galactose (Gal), and N-acetylgalactosamine (GalNAc) (Schnaar and Kinoshita, 2015). The most common globosides are globotriosylceramide (αGal14βGal14βGlc1-Cer, Gb3) and globotetraosylceramide (βGalNAc13αGal14βGal14βGlc1-Cer, Gb4).

**FIGURE 1:**
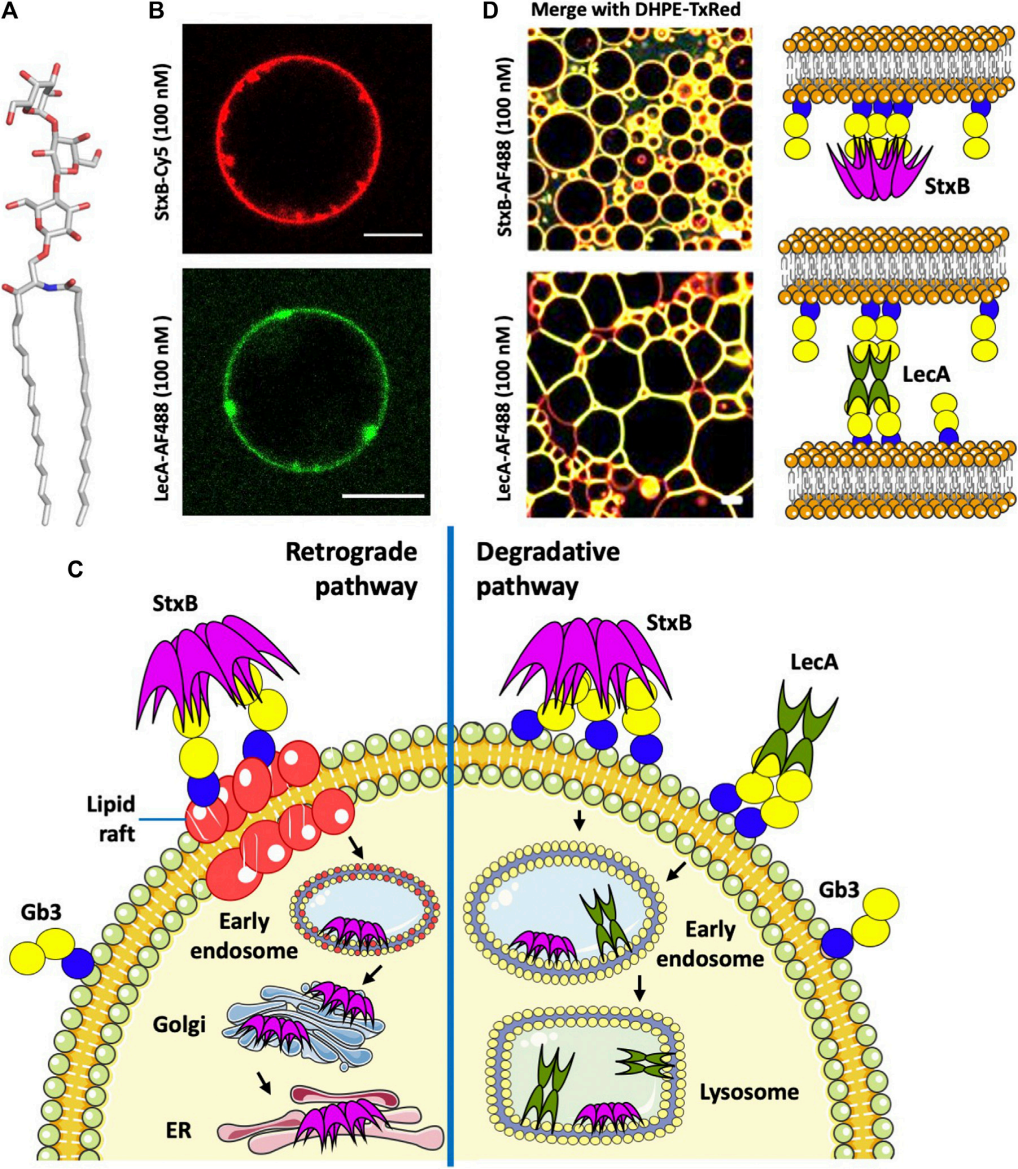
**(A)** Structure of the glycosphingolipid Gb3. **(B)** StxB- and LecA-induced membrane invaginations on Gb3-containing GUVs. **(C)** Endocytotic pathways of StxB and LecA. **(D)** Different effects of lectin topology (LecA and StxB) in proto-tissue (3D assembly of giant liposomes) formation (Parts of panel D adapted from image by Sarah Villringer and licensed under CC BY 4.0; https://doi.org/10.1038/s41598-018-20230-6). AF488 (Alexa Fluor 488) and TxRed (Texas Red) are fluorophors, and DHPE-TxRed represents Texas Red linked to the phospholipid 1,2-Dihexadecanoyl- *sn* -Glycerol -3-Phosphoethanolamine. All scale bars are 10 μM, and schematic representations were made using Servier Medical Art (https://smart.servier.com/).

Gb3 is also known as CD77 and P^k^ blood group antigen (Figure 1A). The corresponding carbohydrate epitopes are referred as galabiose for the disaccharide αGal14Gal, and globotriose for the trisaccharide αGal14βGal14Glc. The highest Gb3 amount is present in human glomerular microvascular endothelia and the proximal tubule cells of the kidney (O’Loughlin and Robins-Browne, 2001). Other Gb3 presenting cells include the colonic microvascular endothelia (Jacewicz et al., 1999) and the endothelial vasculature of the cerebellum (Ren et al., 1999). Also, Gb3 is expressed in B-cells (Mangeney et al., 1991).

The biosynthesis of Gb3 is catalysed by the Gb3 synthase, an α1,4-galactosyltransferase encoded by the *A4GALT* gene, via the transfer of galactose to lactosylceramide acceptor. Interestingly, the same enzyme synthesizes the P1 antigen (Iwamura et al., 2003; Thuresson et al., 2011). The degradation of Gb3 is performed by α-galactosidase (GLA), cleaving the α-galactose. Deficiency of GLA hydrolase leads to the intra-lysosomal accumulation of undegraded Gb3, causing Fabry disease, which predominantly affects the central nervous system (CNS), heart, and kidney (Bekri et al., 2006).

In humans and other mammals, the αGal14Gal epitope was considered to be present only on glycolipids (Gb3 and P1 antigen), but it was recently demonstrated that the Gb3 synthase can also produce αGal14Gal-capped N-glycans in transfected CHO cells (Szymczak-Kulus et al., 2021). This epitope is widely present on N-glycoproteins in birds with substantial similarity between pigeon α4GalT and human Gb3 synthase (72.5%) (Suzuki et al., 2004). The αGal14Gal epitope is also present on O-glycans in some birds and amphibians (Suzuki, 2019). This comprehensive review concludes that most species of mammals possess an active Gb3 synthase, while putative α4GalT is present in all vertebrates, with proven activity in birds and some amphibians. Recent analysis of genomes indicated potential members of this enzyme family also in plants and insects, but with no information on the specificity of these enzymes that can reflect the wide α-glycosyltransferase activity of family GT32 (Keusch et al., 2000).

Gb3 is present in the extracellular leaflet of the plasma membrane and plays a significant role in microbial attachment to the host cell surface. Furthermore, Gb3 is a tumour-associated GSL, highly present in a plethora of human cancers, including breast cancer and lymph node metastases (LaCasse et al., 1999; Stimmer et al., 2014), Burkitt’s lymphoma (Mangeney et al., 1993), ovarian (Jacob et al., 2012), colorectal (Kovbasnjuk et al., 2005) and pancreatic cancer (Maak et al., 2011). Gb3 is also associated with multidrug resistance as it functionally interplays with the ABC membrane efflux transporter - MDR1 gene in drug-resistant cancers (Mattocks et al., 2006; De Rosa et al., 2008). Gb3 is essential in both human health and disease, and specific Gb3-binding lectins have a high potential in therapeutical approaches. There is a considerable need to investigate the most specific ones.

Gb3 is mainly partitioning in lipid rafts, which are membrane domains enriched in sphingomyelin and cholesterol. The degree of unsaturation, chain length (Kiarash et al., 1994), hydroxylation (Binnington et al., 2002), and heterogeneity (Pellizzari et al., 1992) of Gb3 fatty acyl chains can affect the lateral lipid mobility in the plasma membrane and influence the conformation of the trisaccharide head group on the cell surface. The nature of the fatty acyl chain of Gb3 also influences the binding of receptors such as Shiga toxin, as discussed below (Schütte et al., 2014; Schütte et al., 2015; Schubert et al., 2020).

## Gb3-dependent Binding and Uptake Strategies

Several pathogens and pathogen toxins hijack Gb3 at the cell surface for adhesion, and in several cases, also for internalization. The examples of Shiga toxin and the bacterium *P. aeruginosa* are discussed below.

### Shiga Toxin–Binding, Internalization, and Toxicity

Members of the Shiga toxin (Stx) family are structurally and functionally related proteins belonging to AB_5_ holotoxins produced by pathogenic bacteria (Fan et al., 2000). The primary toxin-producing bacterium, *Shigella dysenteriae*, and the toxin itself were named after the Japanese bacteriologist Kiyoshi Shiga, the first to describe and isolate the bacterium in 1897 (Keusch et al., 1995; Trofa et al., 1999). About eighty years later, Shiga toxin-producing *Escherichia coli* (STEC) strains appeared to also cause diarrhea, like *S. dysenteriae*. Two types of immunologically distinct toxins have been identified: Shiga toxin type 1 (Stx1), which is very similar to Shiga toxin produced by *S. dysenteriae,* and type 2 (Stx2) (Konowalchuk et al., 1977). However, independently from the bacterial origin and the mode of action, these toxins are generally called Shiga toxins (Scheutz et al., 2012).

All Shiga toxins consist of a catalytically active A-subunit and a homopentameric, receptor-binding B-subunit. The A-subunit is composed of A1-and A2-domains linked *via* a disulfide bond. When an intracellular protease releases the A1-domain from A2, it becomes catalytically active and is classified as a Type II ribosome-inactivating protein (RIP). Inhibition of protein synthesis by Shiga toxin is sufficient to kill a host cell when properly processed and delivered. There are subtle differences between types (Stx1 and Stx2) and subtypes (genetic variants) of Shiga toxins in terms of specificity and binding strength (Paton et al., 2004; Scheutz et al., 2012).

Shiga toxin enters the cells after binding to its receptor, the glycosphingolipid Gb3, on the host cell surface. The B-subunit of Shiga toxin (StxB) is able to induce tubular membrane invaginations (Figure 1B), as demonstrated in host cells and giant unilamellar vesicles (GUVs), to initiate its cellular uptake (Römer et al., 2007; Römer et al., 2010). Endocytosis of Shiga toxin (Figure 1C) is either clathrin-dependent or, to a lesser extent, clathrin-independent (Sandvig et al., 1992). Also, cytoskeletal dynamics is required for endocytosis in human kidney cells (Torgersen et al., 2007). After endocytosis, Shiga toxin follows either the degradative pathway to lysosomes when bound to non-lipid raft Gb3, or is transported retrograde to the Golgi apparatus and ER when bound to Gb3 associated with lipid rafts (Figure 1C). Binding to lipid rafts is critical for the toxicity of Shiga toxin, as retrograde transport to the cytosol facilitates toxicity, while endosome sorting to lysosomes targets the toxin for degradation hence decreasing toxicity (Sandvig et al., 1992; Sandvig et al., 2010). For instance, domestic cattle are not affected by Shiga toxin poisoning because all toxin bound to intestinal epithelial cells is transported to lysosomes for degradation (Hoey et al., 2003). In addition of the saccharide itself, the membrane environment and cholesterol levels are essential parameters for Shiga toxin binding and toxicity (Kiarash et al., 1994; Arab and Lingwood, 1996; Nakajima et al., 2001; Mahfoud et al., 2009; Schubert et al., 2020).

### *P. aeruginosa* Lectin LecA – Role in Cellular Uptake of the Bacterium

*Pseudomonas aeruginosa* is an opportunistic Gram-negative bacterium colonizing different human tissues and responsible for lung infections in cystic fibrosis and immune-compromised patients, especially in hospital environment (Eberl and Tummler, 2004). This bacterium causes chronic and acute pneumonia, dermatitis, wound and burn sepsis, and also impairs the wound healing process. Currently, there is a high need for new therapeutics to limit its spread and mode of action (Wagner et al., 2016). Among many virulence factors and antibiotic resistance determinants (Jurado-Martin et al., 2021), this pathogen produces two tetrameric lectins, LecA and LecB (also known as PA-IL and PA-IIL), with specificity to terminal α-D-galactose (αGal) and L-fucose (Fuc), respectively (Gilboa-Garber, 1982). LecA binds very efficiently to Gb3-containing GUVs. Besides its ability to induce membrane invaginations that appear rather different to StxB-induced membrane tubules (Figure 1B), it can crosslink liposomes (Figure 1D), in contrast to StxB, probably due to the different orientations of Gb3 binding sites in both lectins. LecA binding induces elongated proto-cellular junctions, which shape the vesicles into polygonal clusters resembling cellular tissues (Villringer et al., 2018).

LecA has been shown to play a crucial role in the internalization of the whole bacterium into host cells. LecA binding and clustering of Gb3 induces negative membrane curvature (Kociurzynski et al., 2021) resulting in the membrane engulfment of the bacterium via the “lipid zipper” mechanism (Eierhoff et al., 2014). Experiments on several epithelial cell lines confirmed the dependence of this internalization on the presence of both LecA at the bacterial outer membrane and Gb3 in the host cell membrane. LecA alone does not follow the retrograde transport route, but rather traffics to late endosomes and lysosomes in epithelial cells (from personal communication with Annette Brandel) (Figure 1C), and through apical recycling endosomes in polarized cells (Müller et al., 2017). LecA toxicity has been demonstrated in primary respiratory epithelial cells and in a mouse model of gut-derived sepsis. LecA decreased the percentage of activated ciliated cells (Bajolet-Laudinat et al., 1994), probably by permeabilizing the epithelial cells, permitting a much more decisive action of associated toxins (Laughlin et al., 2000). Inhibiting LecA by galactose or galactose-derived compounds proved to be efficient against lung infection in murine model systems (Chemani et al., 2009; Boukerb et al., 2014). Furthermore, LecA selectively bound to cardiac non-myocytes and altered plasma membrane topology (Darkow et al., 2020).

When incubated simultaneously with cells, LecA and StxB localize to different membrane nanodomains, despite binding to the same carbohydrate epitope. Moreover, StxB stains the primary cilium, which LecA does not (Schubert et al., 2020). It is therefore of interest to analyse the structural differences between these two Gb3-receptors.

## Diversity, Structural Organization and Binding Mechanisms of Gb3-Binding Lectins

**FIGURE 2:**
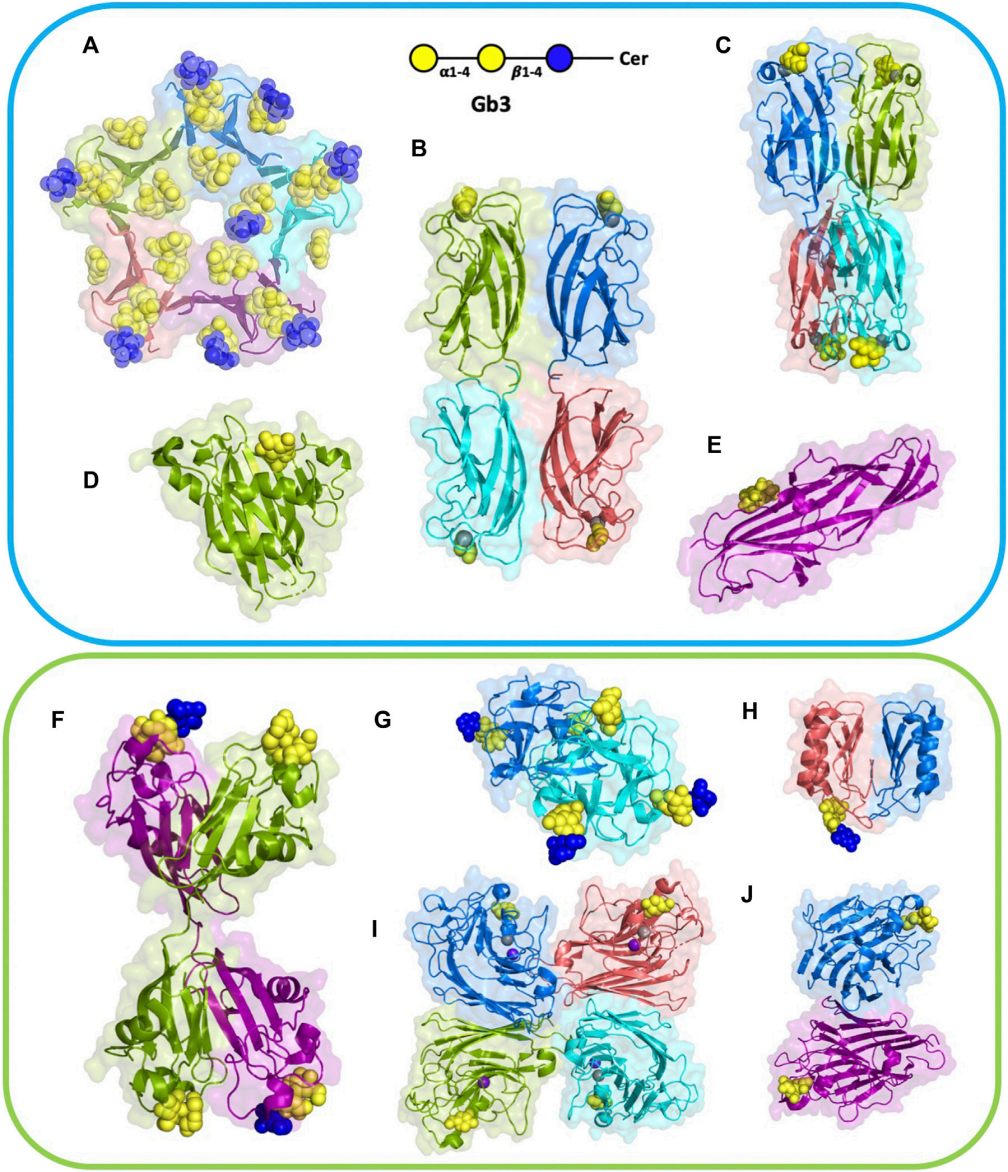
Structures of selected lectins complexed with Gb3. Microbial lectins (light blue frame): **(A)** homopentameric B-subunit of Shiga toxin (StxB) from *Shigella dysenteriae*/*Escherichia coli* (1BOS); **(B)** homotetrameric LecA from *Pseudomonas aeruginosa* (2VXJ); **(C)** homotetrameric PIIA from *Photorhabdus luminescence* (5ODU), a LecA homolog; **(D)** monomeric SadP adhesin from *Streptococcus suis* (5BOA); **(E)** PapG monomer from *Escherichia coli* (4Z3H); Other Gb3-binding lectins (light green frame): **(F)** homodimeric CSL3 from *Oncorhynchus keta* (2ZX4). **(G)** homodimeric CGL lectin from *Crenomytilus grayanus* (5F90); **(H)** homodimeric LDL from *Lyophyllum decastes* (4NDV); **(I)** Jacalin heterotetramer from *Artocarpus integrifolia* (2ZMK); **(J)** homodimeric WBA-I lectin from *Psophocarpus tetranoglobus* (2ZMK). Each monomer is colored differently, either in deep salmon, purple, cyan, marine, or split pea color. Galactose residues are shown in yellow and glucose in blue color; calcium is represented by grey sphere and manganese by deep purple sphere. The 3D representations were visualised using PyMol (https://pymol.org/2/).

Several lectins from different origins have specificity for Gb3 carbohydrate moiety. Structural data are available for many of them, and searching in the Unilectin3D database (Bonnardel et al., 2019) results in 15 complexes of lectins with the αGal14Gal disaccharide, or oligosaccharides with this terminal epitope. Six structures involve Shiga toxin (Stx) from *S. dysenteriae* and *E. coli*, but other proteins, such as bacterial adhesins and soluble lectins from plants, fungi, and fish, have also been crystallized with this epitope (Figure 2; Table 1). The lectin LecA from *P. aeruginosa* that also binds to Gb3 has been crystallized with other αGal containing oligosaccharides and is also included in this structural review.

**TABLE 1:** Gb3-binding lectins and their specifications. The number of sites is indicated per functional oligomeric assembly. The specificity (evaluated as star numbers) indicates the preference for Gb3 oligosaccharides over other Gal-containing ones. PDB code is indicated for complexes with Gb3 oligosaccharides (except LecA and PIIA complexed with αGal).

Lectin	Origin	Species	Fold	Kd	Sites	Specificity	PDB code
STXB	Bacteria	*Escherichia coli*	OB-fold	0.5–1 mM ITC^a^	15	***	1BOS 1QNU
LECA	Bacteria	*Pseudomonas aeruginosa*	β-sandwich	77 µM ITC^b^	4	**	1OKO (αGal)
PIIA	Bacteria	*Photorhabdus luminescens*	β-sandwich	1 mM IC_50_ ^c^	4	**	5ODU (αGal)
PAPG	Bacteria	*Escherichia coli*	β-sandwich	1.16 mM ITC^d^	1	**	4Z3G 4Z3H
SADP	Bacteria	*Streptococcus suis*	a/β mixed	3 µM ITC^e^	1	***	5BOA
LDL	Fungi	*Lyophyllum decastes*	knottin	0.38 mM ITC^f^	2	**	4NDV
CGL	Animal	*Crenomytilus grayanus*	β-trefoil	14 µM BLI^g^	6	**	5F90
CSL3	Animal	*Oncorhynchus keta*	β-sandwich	26 µM FAC^h^	4	***	2ZX4
JACALIN	Plant	*Artocarpus integrifolia*	β-prism	1.2 mM ITC^i^	4	*	5J51
WBA-I	Plant	*Phosphocarpus tetranoglobus*	β-sandwich	0.67 mM ITC^j^	2	*	2ZMK 2ZML

a αGal14βGal14Glc trisaccharide, determined by isothermal titration calorimetry (ITC) (St Hilaire et al., 1994).

b αGal14βGal14Glc trisaccharide, determined by ITC (Blanchard et al., 2008).

c αGal14Gal disaccharide, determined as IC50 by fluorescence polarization (Beshr et al., 2017).

d αGal14Gal disaccharide, determined by ITC (Navarra et al., 2017).

e αGal14Gal disaccharide, determined by ITC (Zhang et al., 2016).

f αGal14βGal14Glc trisaccharide, determined by ITC (Goldstein et al., 2007).

g αGal14βGal1βGlc-allyl derivative, determined by biolayer interferometry (BLI) (Liao et al., 2016).

h αGal14βGal14Glc trisaccharide, determined by Frontal Affinity Chromatograpy (FAC) (Watanabe et al., 2009).

i αGal14Gal disaccharide, determined by ITC (Mahanta et al., 1990).

j αGal14Gal disaccharide, determined by ITC (Puri and Surolia, 1994).

Gb3-binding lectins appear to be structurally diverse proteins with no sequence similarity, and varying size and architecture. They also vary in oligomerization number, orientation, and depth of sugar-binding pockets, and the binding modes to the exposed oligosaccharide on the cell membrane, which is also sensitive to the membrane environment.

### Structural Analysis of Shiga Toxin – An AB_5_ Bacterial Holotoxin

**FIGURE 3:**
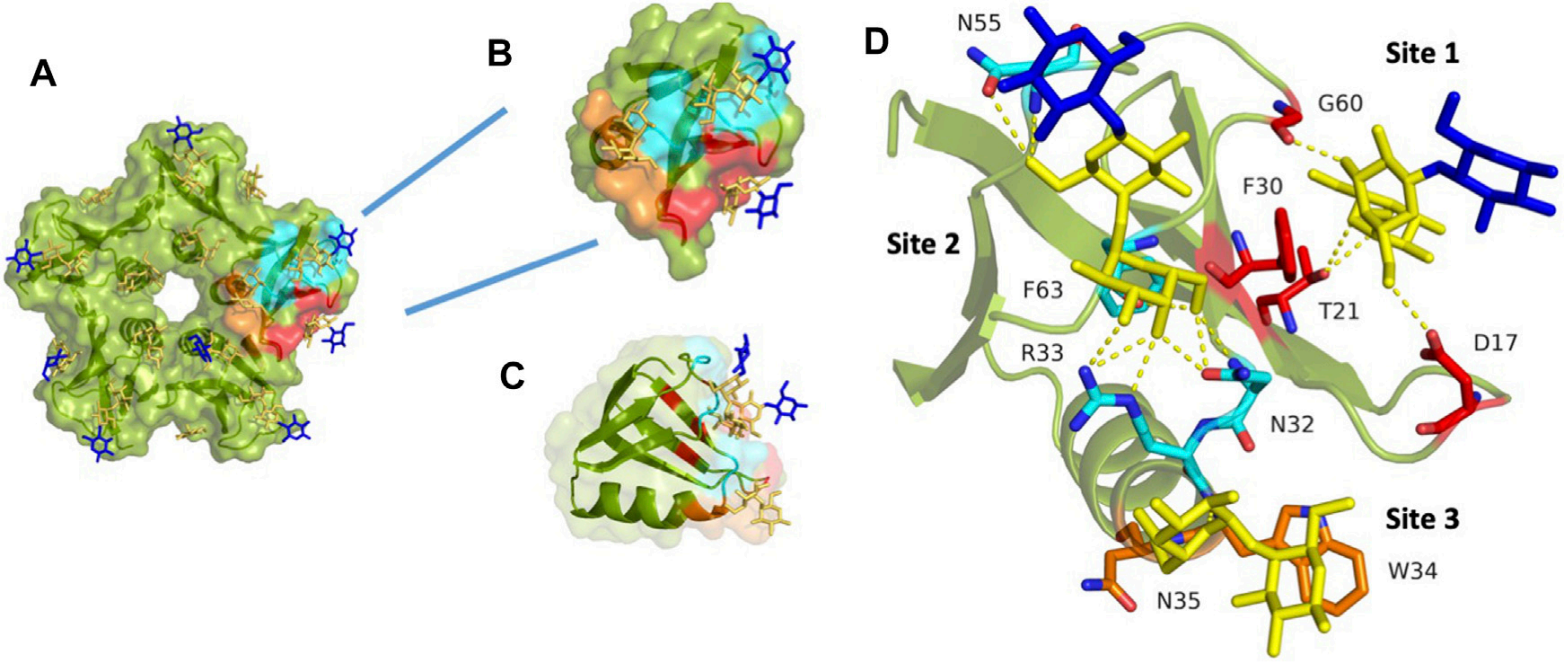
StxB contains three Gb3 binding sites per monomer. **(A)** The overall monomer representation of the Gb3 binding sites in the StxB pentamer from *Shigella dysenteriae*/*Escherichia coli* (PDB id:1BOS); **(B)** Overview of the monomeric structure of StxB with three Gb3 binding sites; **(C)** The monomeric structure of StxB demonstrating the OB-fold; **(D)** Zoomed Gb3 binding sites of StxB. Galactose residues are yellow and glucose are in blue color. Hydrogen bonds are shown in yellow dash lines. The 3D representations were visualised using PyMol (https://pymol.org/2/).

Shiga toxin consists of a pentamer of five identical B-fragments (forming the B-subunit, StxB) associated with the enzymatic A-subunit (StxA). StxB is responsible for holotoxin binding to receptors, such as Gb3, on the host cell surface (Figures 2A, 3A). The B-subunit (Figure 3B) adopts the ubiquitous oligonucleotide/oligosaccharide-binding fold (OB-fold). The OB-fold can bind oligonucleotides, proteins, metal ions, catalytic substrates, and oligosaccharides (Murzin, 1993). It is comprised of a five-stranded antiparallel β-barrel, which is also present in other AB_5_ toxins, such as cholera toxin or pertussis toxin, but with different oligosaccharide specificity and no similarity in amino acid sequence. In the case of StxB, one end of the barrel is capped by an α-helix (Figure 3C). This type of topology resembles a Greek key and is known as a closed β-sheet. Although OB-fold proteins like the heat-labile LTB, the Shiga toxin family, and the yeast aspartyl-tRNA synthase (AspRS) share no sequence homology, α-helices have very similar orientations and can be easily superimposed (Murzin, 1993). Both types of Shiga toxins, type 1 (Stx1) and type 2 (Stx2), are structurally similar, but differ in amino acid sequences (Scheutz et al., 2012).

When the A-subunit is absent, the StxB still adopts the same pentameric structure as when the holotoxin binds the host receptors (Donohue-Rolfe et al., 1989). All of the Stx1 and most of the Stx2 toxins bind exclusively to Gb3. However, Stx2f and Stx2e can bind Gb4 in addition to Gb3 (DeGrandis et al., 1989; Skinner et al., 2013). Thus, slight peptide sequence differences influence the carbohydrate-binding specificity of Shiga toxins. Interestingly, a new subtype, Stx2k, has been recently discovered, which is very similar to, but much less toxic than Stx2a (Hughes et al., 2019).

There are fifteen potential binding sites per Shiga toxin pentamer since each B-monomer has three sugar-binding sites, as shown in Figures 3A,B. Stx1B and Stx2B are exhibiting a dissociation constant (Kd) range of 0.5–1 mM for the B-subunit monomer toward globotriose. Some differences in published dissociation constants appear from various studies measuring Kd values with different techniques and varying sensitivity (Head et al., 1991; Ling et al., 1998; Soltyk et al., 2002; Flagler et al., 2010).

Stx1 interacts exclusively with the carbohydrate moiety of Gb3, while Stx2 needs additional interactions with the full glycolipid (Gallegos et al., 2012). This necessity has also been recently demonstrated in studies using the P1 glycotope, which was N-linked to the synthetic membrane protein Saposin D. This synthetic receptor mediated Stx1 entry into cells, but not the uptake of Stx2 (Szymczak-Kulus et al., 2021). Furthermore, Stx1 and Stx2 prefer to bind to Gb3 containing an α-hydroxyl fatty acyl chain, but not to a Gb3 analog without the hydroxyl group (Binnington et al., 2002). Albeit Stx1 and Stx2 bind the same glyco-receptor, Gb3, they slightly bind differently. For instance, in a solid-phase Gb3 ELISA system, preincubation with Stx1 blocks the subsequent Stx2 binding, but not *vice versa* (Itoh et al., 2001). It seems that Stx1 demonstrates faster binding kinetics when compared to Stx2, but once Stx2 is bound, it is more difficult to remove since it binds stronger (Nakajima et al., 2001).

Looking closer at the carbohydrate binding sites of Stx1B (Figure 3D), it was known, for a long time, that there is almost no binding to Gb3 by the Stx1B double mutant D16H/D17H in site #1 (Jackson et al., 1990). Asp17 forms a hydrogen bond with Gal2 (penultimate galactose moiety) in site #1. However, Asp16 is involved in Gb3 binding in site #2 (Ling et al., 1998). For site #1, the stacking interaction with Phe30 is typical in carbohydrate-binding proteins. Also, Phe30 might be as crucial in binding as Trp34 in site #3, as both amino acids are aromatic (Ling et al., 1998).

Moreover, the B-subunits of Stx2e (Tyrrell et al., 1992) as well as Stx2f (Skinner et al., 2013) and Stx2k (Hughes et al., 2019) are also known to bind Gb4, in addition to Gb3. It was also shown that the double mutant (Q65E/K67Q) of Stx2e altered back the binding preference from Gb4 to Gb3 (Tyrrell et al., 1992).

Numerous hydrogen bonds binding Gal1 (terminal galactose moiety) dominate at binding site #2. However, stacking interaction is not present at this site. As mentioned above, Asp16 and Thr62 are essential in binding to Gb3 oligosaccharide. There are fewer contacts with Gb3 at site #2 than at other sites. This is due to the perpendicular position of StxB to the receptor surface. The D18N mutation retains binding activity to Gb3 and Gb4 for the B-subunit of Stx2e *via* hydrogen bonds. The different faces of aromatic Trp34 interact with Gal1 and Gal2 (Ling et al., 1998). A new serotype, the Stx2k, has been recently discovered. It is less toxic than Stx2a but is similar in receptor-binding preference. However, there are two amino acid differences in the receptor-binding site #1 and site #2, which might explain differences in cytotoxicity (Hughes et al., 2019).

### LecA and Other Related Bacterial Soluble Lectins

The lectin LecA from *P. aeruginosa* is a tetramer (Figure 2B), each unit consisting of 121 amino acids (12.75 kDa) (Gilboa-Garber, 1982). The crystal structure depicts one calcium-dependent galactose binding site per monomer (Cioci et al., 2003). Each monomer possesses a β-sandwich jelly-roll fold consisting of two curvy sheets with four anti-parallel β-strands.

**FIGURE 4:**
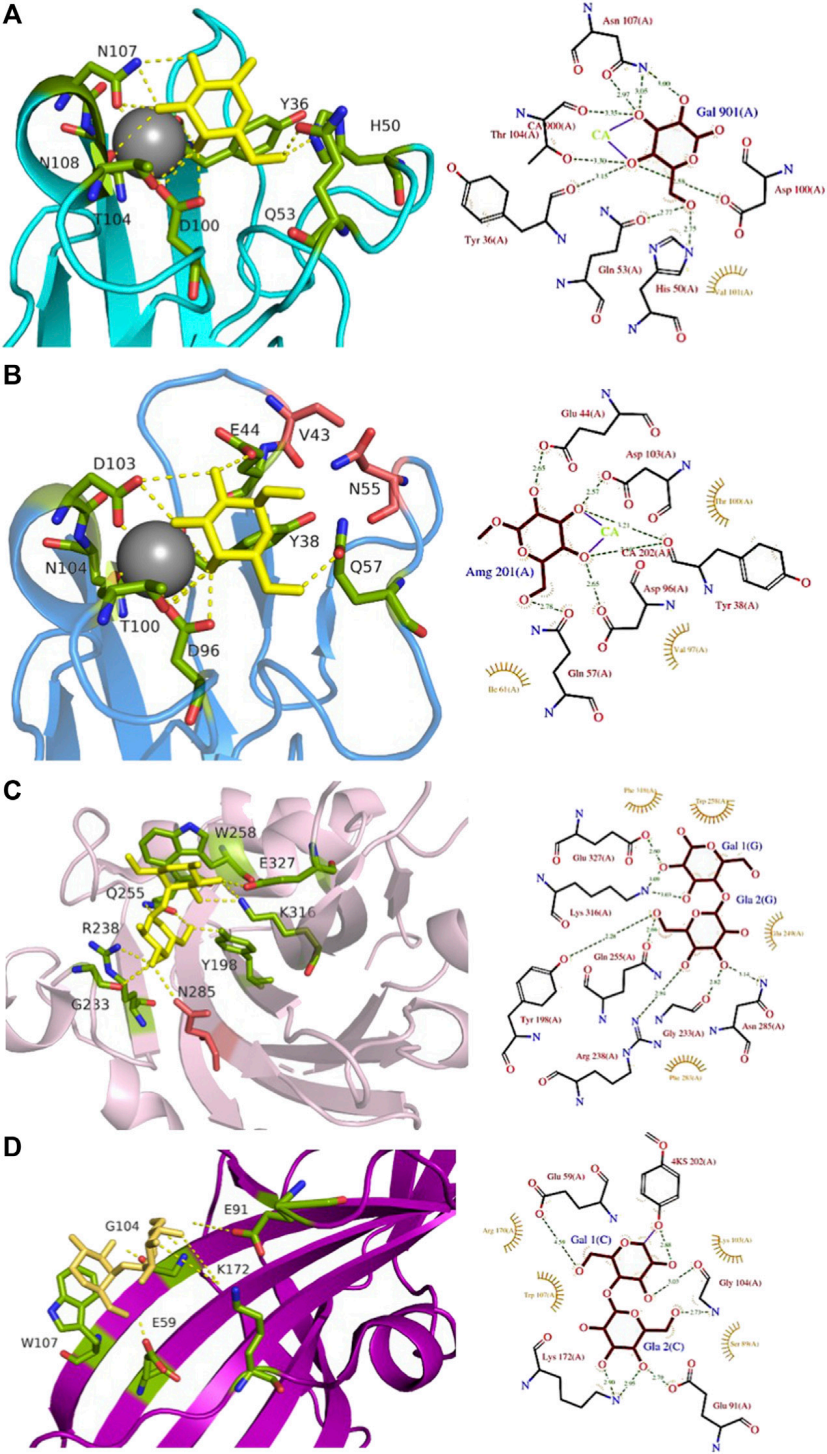
Comparison of Gb3-binding sites of LecA, PIIA, PapG, and SadP: **(A)** LecA from *Pseudomonas aeruginosa* (1OKO); **(B)** PIIA from *Photorhabdus luminescens* (5ODU); **(C)** SadP adhesin from *Streptococcus suis* (5BOA); **(D)** PapG adhesin from *Escherichia coli* (4Z3G). Galactose residues are shown in yellow and calcium is represented by a grey sphere. Hydrogen bonds are shown in yellow dash lines. The 3D representations were visualised using PyMol (https://pymol.org/2/), and 2D ligplot schemes were done using PDBsum.

LecA from *Pseudomonas aeruginosa* binds to α-galactosylated lipids and proteins on animal tissue (Kirkeby et al., 2006). It recognizes efficiently the disaccharides αGal14βGal and αGal13βGal (Chen et al., 1998), Binding to sphingolipids capped with αGal14βGal and αGal13βGal was confirmed by thin-layer chromatography (Lanne et al., 1994). LecA agglutinates erythrocytes with Gb3 (blood group P^k^), but also with blood group B and blood group P1 (Gilboa-Garber et al., 1994), and binds strongly to Burkitt lymphoma cells that present large amounts of the globotriaosylceramide antigen Gb3/CD77/P^k^ (Blanchard et al., 2008). The crystal structure of the complex of LecA with iso-Gb3 trisaccharide revealed how the penultimate galactose interacts with the protein surface, rationalizing the high affinity (Blanchard et al., 2008). A crystal structure with Gb3 oligosaccharide could not be obtained, but molecular modelling demonstrated that other contacts are established for this oligosaccharide (Figure 4A). Furthermore, the firm binding observed when Gb3 is inserted in the membrane, such as on Burkitt Lymphoma cells, was rationalized since the Gb3 glycosphingolipid geometry promotes the parallel presentation of neighbouring trisaccharide heads, fully compatible with multivalent binding by LecA. Further modelling of the binding of LecA with Gb3 embedded in the extracellular leaflet of a lipid bilayer confirmed the likely clustering of αGal14βGal by this lectin (Kociurzynski et al., 2021).

LecA binds to galactose, galabiose and other αGal-containing oligosaccharides with medium affinity (Kd in the range of 50 µM) (Blanchard et al., 2008), although with a slight preference for melibiose, the αGal16Glc epitope present in raffinose and other plant oligosaccharides (Chen et al., 1998). It does not recognize lactose and other βGal-containing oligosaccharides since His50 would create a steric conflict with the second residue, but the presence of an hydrophobic pocket on the protein surface close to the anomeric position of galactose provides higher affinity for β-galactose functionalized with aromatic ring (Rodrigue et al., 2013). LecA has four binding sites, and due to its topology, two are associated side by side in perfect orientation for binding to Gb3 in membrane (Kociurzynski et al., 2021). This results in avidity that is much stronger than local affinity, with values in the nM range when evaluated with multivalent ligands (Bernardi et al., 2013).

A LecA homolog, PIIA (Figure 2C) has been identified in *Photorhabdus luminescens,* another Gram-negative bacterium, presenting a complex interaction cycle with nematodes and insects (Clarke, 2020). The PIIA lectin shares 37% sequence identity to LecA and presents the same range of affinity for α-galactosylated epitopes (Figure 4B) (Beshr et al., 2017).

### Bacterial Adhesins SadP and PapG

Pathogenic bacteria present various adhesins on their surface to bind to host tissue or various surfaces (Viela et al., 2020). Some of these adhesins, present on flagella, or different types of pili, have carbohydrate-binding domains with specificity to host tissues.

*Streptococcus suis* is responsible for infection in pigs and acts as an opportunistic human pathogen responsible for two outbreaks in China. Among the 35 identified serotypes, some are causing severe pneumonia and sepsis in swine, and meningitis in humans and swine (Gottschalk et al., 2010; Goyette-Desjardins et al., 2014). The streptococcal factor H-binding protein is also an adhesin (SadP) and contributes to zoonotic transmission by binding to both human and porcine intestinal epithelial cells (Ferrando et al., 2017). SadP (Figure 2D) is a monomeric 80 kDa adhesin anchored in the bacterial cell wall. Characterization of its binding specificity showed that SadP recognizes αGal14βGal containing oligosaccharides (Figure 4C) and binds to Gb3 through its N-terminus (Kouki et al., 2011). The binding of SadP to Gb3 is one of the crucial steps for the bacterium to pass the blood-brain barrier and access the central nervous system resulting in meningitis development (Kong et al., 2017).

The crystal structure of the N-terminal galabiose-binding domain reveals that SadP adopts a β-sandwich core domain composed of three α-helices and ten β-strands (Zhang et al., 2016). The crystal structure of SadP complexed with galabiose demonstrates the balance of hydrogen bonds and hydrophobic interactions for the terminal α-galactoside (Zhang et al., 2016). The affinity for these disaccharides is in the micromolar range measured by isothermal titration calorimetry (ITC) (Kd = 3 µM). Two subtypes of SadP have different fine specificities for glycolipids. The strains with the PN subtype cause meningitis, while the ones with PO, cause asymptomatic carriage and respiratory phenotype (Madar Johansson et al., 2020). Both types of SadP are shown to predominantly bind to Gb3 present in pig lung, but a mutation in the galabiose binding domain of subtype PN strains results in additional binding to globotetraosylceramide (Gb4). The sugar-binding sites in both PN and PO of SadP adhesins are mostly conserved. PO binds to Gb3 with higher affinity than the PN subtype (Kd of 3 µM and of 13 μM, respectively). Interestingly, the mutation from asparagine to aspartate at position 285 (N285D) results in the ability of PN to bind Gb4 with Kd = 34 μM, which is more than 200 times higher compared to the PO subtype (Madar Johansson et al., 2020).

PapG (Figure 2E) is an adhesin present in uropathogenic *E. coli* (UPEC), the leading cause of urinary tract infections in humans. This bacterial adherence factor is located at the tip of P pili, which are composite fibers consisting of a thin tip and a thick pilus rod (Lukaszczyk et al., 2019). PapG, as other tip proteins of chaperone-usher pili (Lukaszczyk et al., 2019), consists of two domains, the N-terminal part is involved in carbohydrate-binding and interacts with the receptor, while the C-terminus is part of the pili architecture and participates in the binding to the chaperone during the biogenesis. PapG mediates the attachment of bacteria to the uroepithelium of the human kidney through binding to the αGal14βGal epitope (Lund et al., 1987; Stromberg et al., 1990). This adhesin adopts a large elongated Ig-like fold composed of eight β-strands connected by long loops and an α-helical section (Dodson et al., 2001; Sung et al., 2001).

The three different classes of PapG, I, II, and III, adhere differently to host-cell glycosphingolipids in the uroepithelial tract with subtle differences in binding specificities (Stromberg et al., 1990). PapG I and PapG II are specific to Gb3 and Gb4, while PapG III prefers to bind Forssman glycosphingolipid (Legros et al., 2019). PapG II adhesin binds weakly to galabiose (Kd approx. 1 mM) but presents a higher affinity for globotriose (Kd = 100 µM) as measured by ITC (Navarra et al., 2017). The structure of PapG II has been obtained in complex with galabiose and with other oligosaccharides of the globoseries (Dodson et al., 2001; Navarra et al., 2017), but in all cases, the main contacts are limited to the αGal14βGal disaccharide moiety (Figure 4D) that forms a dense network of H-bonds and water-mediated interactions involving amino acids in the binding site, as well as stacking of Gal2 with Trp107.

### Gb3-Binding Lectins From Animals, Fungi, and Plants

Many animal organisms, such as fishes and invertebrates, produce various lectins that serve as an innate immunity system. The rhamnose-binding lectins (RBLs) are a family of L-rhamnose or D-galactose binding lectins able to agglutinate various bacteria (Watanabe et al., 2008). Most RBLs are composed of two or three tandem repeats of about 95 amino acids stabilized by four disulfide bridges (Tateno et al., 1998)*.* Three RBLs, CSL1, 2, and 3, from eggs of the chum salmon *Oncorhynchus keta*, bind to Gb3 and induce the production of proinflammatory cytokines (Tateno et al., 1998; Watanabe et al., 2009). CSL3 lectin (Figure 2F) contains two tandem repeated carbohydrate-binding domains with 73% sequence identity, resulting in four binding sites per dimer (Shirai et al., 2009). The overall shape of CLS3 is a kinked dumbbell with four lobes. Each CBD (carbohydrate binding domain) comprises two anti-parallel β-sheets, three α-helices, and four conserved disulfide bonds interconnecting within each domain. More recently, the structure of SUL-I, a RBL from venomous sea urchin *Toxopneustes pileolus*, demonstrated the presence of three tandem lectin domains (Hatakeyama et al., 2017). CSL3 and SUL-I have a similar affinity for the Gb3 trisaccharide (Kd = 26 µM) as determined by frontal chromatography (Watanabe et al., 2009), but SUL-1 is less specific, i.e., binding to a wide range of galactosides (Hatakeyama et al., 2017).

**FIGURE 5:**
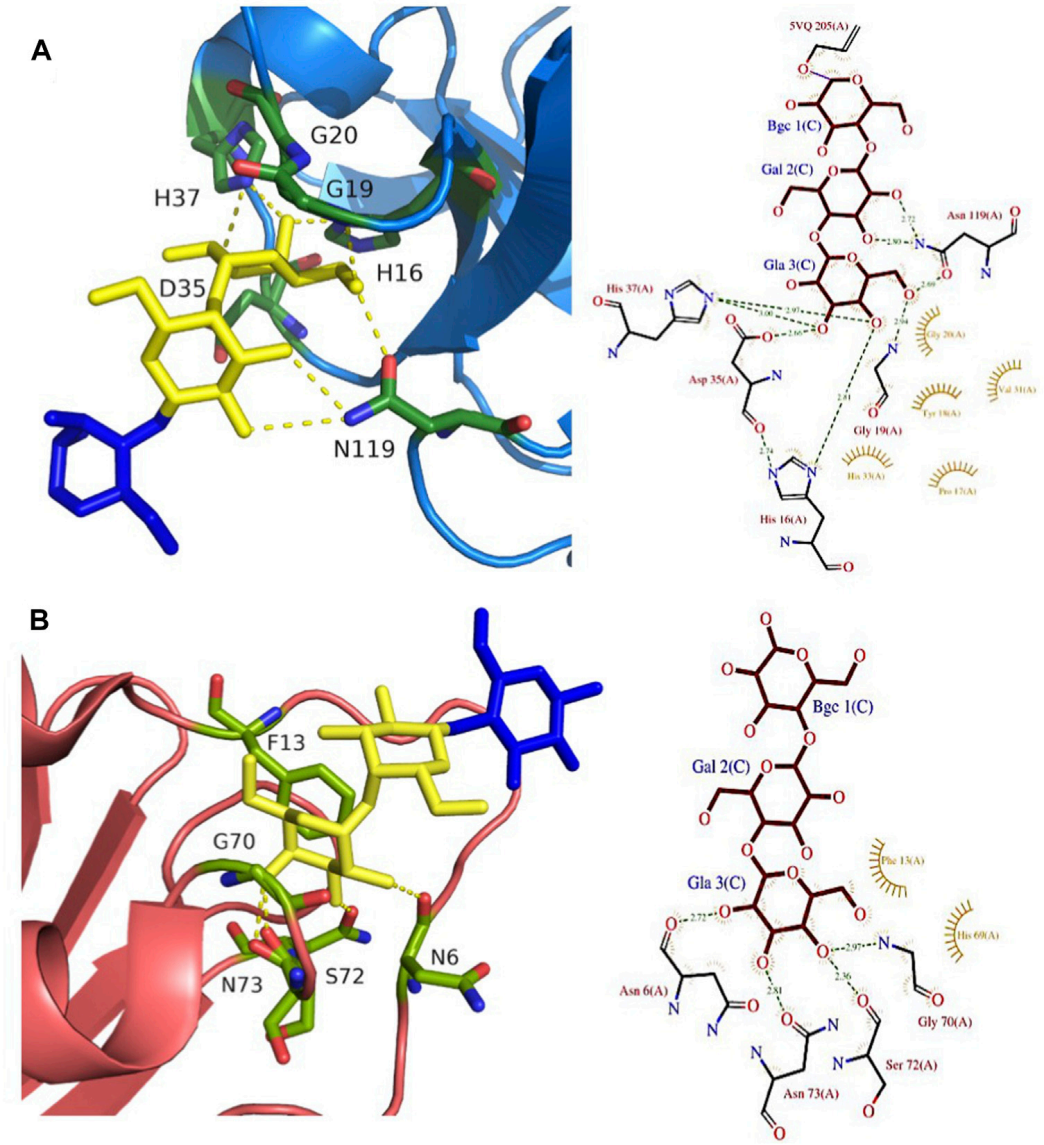
Gb3-binding sites of animal and fungal lectins. **(A)** Animal lectin CGL from *Crenomytilus grayanus* (5F90). **(B**) Fungal lectin LDL from *Lyophyllum decastes* (4NDV). Galactose residues are shown in yellow and glucose in blue color. Hydrogen bonds are shown in yellow dash lines. The 3D representations were visualised using PyMol (https://pymol.org/2/), and 2D ligplot schemes were done using PDBsum.

Another animal lectin family has been purified from bivalves and demonstrated to bind αGal- and αGalNAc-containing oligosaccharides (Belogortseva et al., 1998). Mytilec, a 17 kDa lectin isolated from the Mediterranean mussel *Mytilus galloprovincialis*, binds to globotriose and demonstrates glycan-mediated cytotoxicity towards Burkitt's lymphoma cells (Fujii et al., 2012). The related lectin CGL (Figure 2G) from *Crenomytilus grayanus* also binds to Gb3 on the surface of Burkitt’s lymphoma and breast cancer cells, leading to cell death (Liao et al., 2016; Chernikov et al., 2017). Crystal structures from both lectins demonstrated the occurrence of a dimeric β-trefoil domain, with one galactose binding site in each lobe (Liao et al., 2016; Terada et al., 2016). The structures served as inspiration for engineering Mitsuba-1, a symmetry-constrained β-trefoil with three identical tandem repeats. Mitsuba-1 binds to Gb3-expressing cancer cells, but does not show cytotoxicity (Terada et al., 2017). CGL has a highly conserved carbohydrate-binding motif in the three lobes, consisting of two His, one Gly, and one Asp (Figure 5A). CGL binds to an allyl derivative of globotriose with high affinity (Kd = 14 µM) as determined by biolayer interferometry (Liao et al., 2016). The specificities of MytiLec and CGL are extended to other αGal containing oligosaccharides, including αGal13βGal14Glc (iso-Gb3 trisaccharide) and αGal16Glc (Liao et al., 2016; Terada et al., 2017).

Fungi express a large variety of lectins that are considered as a defence against feeders and pathogens. LDL (Figure 2H) is a 10 kDa αGal binding lectin isolated from *Lyophyllum decastes* fruiting bodies with no sequence similarities with other fungi lectins (Goldstein et al., 2007). The crystal structure demonstrated a homodimer assembly, with monomers consisting of a cysteine-knottin fold made of five-stranded anti-parallel β-sheet and two closely packed α-helices (van Eerde et al., 2015). The carbohydrate-binding pocket is well defined (Figure 5B) and is relatively deep and narrow with binding orientation at the same face for the homodimer. The affinity for galabiose is relatively weak in the millimolar range, but the specificity is high. It should be noted that LDL binds to the Gb3 oligosaccharide, but not efficiently to the glycosphingolipid Gb3, so it can be used only as a probe for terminal galabiose of non-lipidic nature (van Eerde et al., 2015).

**FIGURE 6:**
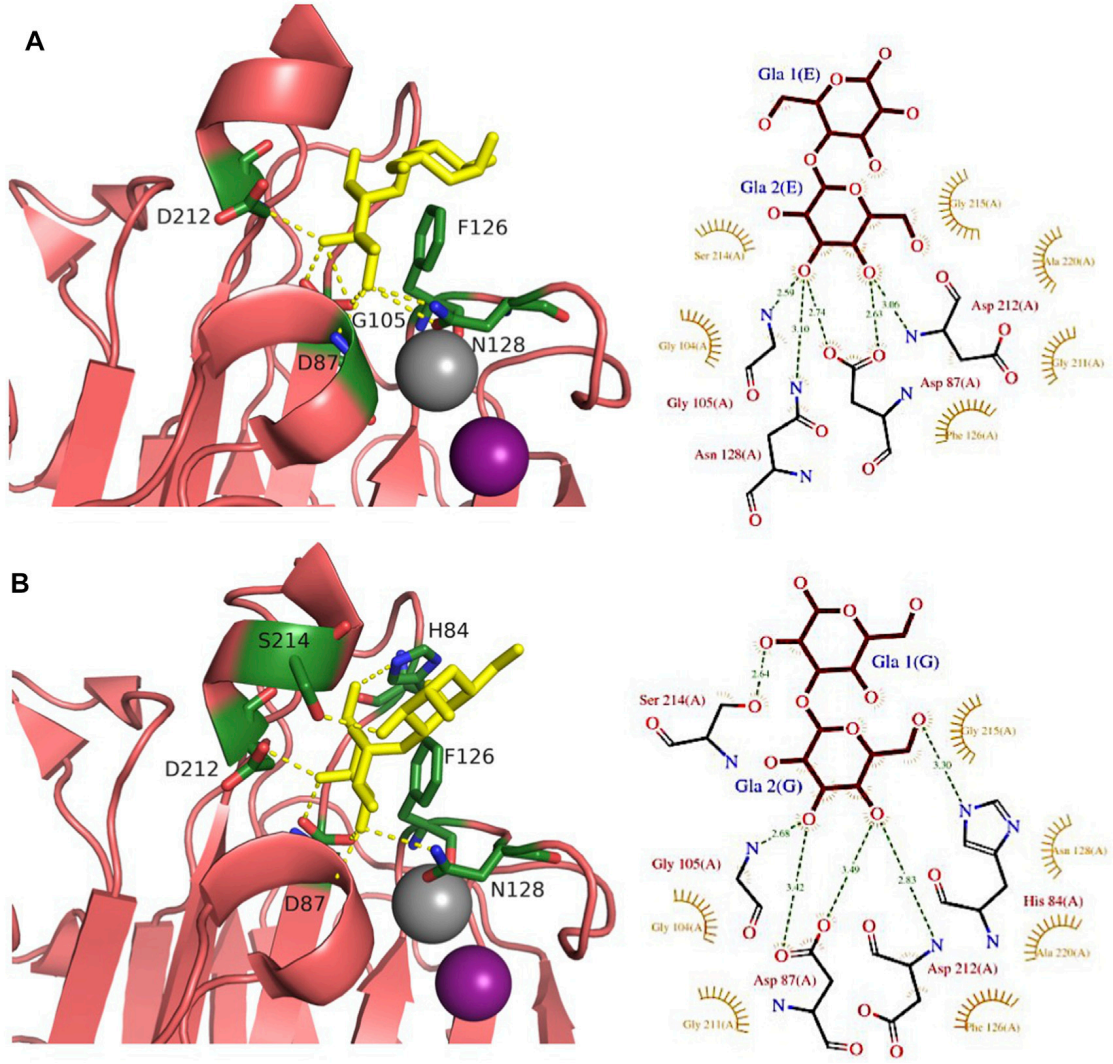
Comparison of αGal14Gal and αGal13Gal binding sites of WBA-I lectin from *Psophocarpus tetranoglobus*. **(A)** WBA-I bound to αGal14Gal (2ZML). **(B)** WBA-I bound to αGal13Gal (2E53). Carbohydrate ligands are shown as yellow sticks. Calcium is represented in grey and manganese in deep purple spheres. Hydrogen bonds are shown in yellow dash lines. Proteins are represented by ribbon with amino acids interacting with ligand as sticks. The 3D representations were visualised using PyMol (https://pymol.org/2/), and 2D ligplot schemes were done using PDBsum.

A large number of plant lectins have been characterized due to their availability in large quantities and their utility in biotechnology. Some of them bind to Gb3 epitope, but the specificity is weak. Jacalin (Figure 2I) from *Artocarpus integer* is a β-prism lectin that has been crystallized with galabiose (Abhinav et al., 2016). The lectin has a promiscuous specificity towards all αGal-containing oligosaccharides. Similarly, winged bean agglutinin (WBA-I) from *Psophocarpus tetragonolobus* (Figure 2J) has been characterized in complex with galabiose (Kulkarni et al., 2008), but the specificity of the lectin is directed towards all galactosides. The structural bases for recognition of αGal14βGal and αGal13βGal are represented in Figure 6.

## Conclusion

In conclusion, a large variety of sequences and folds have been observed for Gb3-binding lectins illustrating the convergent evolution for recognition of this epitope. A frequent pattern is the prevalence of β-sheets in the carbohydrate-binding domain, as observed for StxB, LecA, PapG, and plant lectins, which is in agreement with the very high representation of this structural motif in lectin structures (Bonnardel et al., 2019). In all binding pockets, several hydrogen bonds are established between the protein and the carbohydrate, and at least one is crucial for binding to the axial O4 group of galactose, participating in the specificity. The amino acid involved is frequently an Arg residue whose side chain can establish two hydrogen bonds, one with O4 and a second one with the neighboring O3 or O6. The presence of Trp or Tyr residues is often observed in binding sites since stacking of the aromatic ring with the apolar face of galactose has a solid stabilizing contribution (Asensio et al., 2013; Hudson et al., 2015). The presence of a bridging calcium ion, as observed for LecA, is less common, but coordination through vicinal axial and equatorial hydroxyl groups (O4 and O3 of galactose) has been observed in other lectins (Imberty and Prestergard, 2017). The common feature for all lectins described here is the multivalency, allowing them to compensate a relatively low affinity at each Gb3-binding site by a strong avidity in the presence of multiple Gb3 molecules, like in the context of lipid rafts.

The variety of fold and binding site of Gb3-binding lectins results in differences in affinity and specificity. These properties of lectins from various origins can be correlated to their hypothetical functions that have to be proposed in view of evolution. The bacterial lectins achieve higher specificity and stronger affinity than the ones from fungi, mollusk, or plants. This could be rationalized by the fact that the optimal recognition of host globosides by pathogens can be seen as the result of co-evolution (Bishop and Gagneux, 2007), resulting in proteins perfectly suited for the binding to these epitopes. On the opposite, lectins from mollusks or fungi are defence proteins, often directed against bacterial polysaccharides. Since many capsular polysaccharides mimic epitopes found in mammalian tissues (Cress et al., 2014), the recognition of glycosphingolipids results from the spatial similarity of glycans present in both bacterial epitopes and human ones.

The structural diversity of lectins described here is inspiring for designing a toolbox of therapeutical proteins that could bind to cancer cells and be used for diagnostics or therapeutical strategies. Indeed, Shiga toxin has been demonstrated as a valuable tool for investigating cellular trafficking, but has also been investigated for its potential for imaging cancer cells or delivering active drugs (Johannes and Römer, 2010; Engedal et al., 2011; Luginbuehl et al., 2018). As exemplified with the design of the Mitsuba-1 artificial lectin (Terada et al., 2017), the availability of variety of scaffolds together with the current development in synthetic glycobiology for engineering of lectin binding sites and multivalency (Hirabayashi and Arai, 2019), open the route for the design of novel Gb3-specific lectins for therapeutical applications.
